# Assessment of Medical Student Research Mentorship in Radiation Oncology

**DOI:** 10.1016/j.adro.2023.101323

**Published:** 2023-07-23

**Authors:** Kristy Bono, Marisa Palmeri, Austin Huang, Jillian R. Gunther, Malcolm D. Mattes

**Affiliations:** a Rutgers New Jersey Medical School, Newark, New Jersey; b Baylor College of Medicine, Houston, Texas; c Department of Radiation Oncology, University of Texas MD Anderson Cancer Center, Houston, Texas; d Department of Radiation Oncology, Rutgers Cancer Institute of New Jersey, New Brunswick, New Jersey

## Abstract

**Purpose:**

Mentored medical student (MS) research opportunities in radiation oncology (RO) provide in-depth exposure to the specialty and may promote greater interest in a career in RO. Many radiation oncologists conduct research; however, the extent to which they directly engage MSs in their research is unknown. The purpose of this study was to characterize MS authorship in American Society for Radiation Oncology (ASTRO) journals.

**Methods and Materials:**

The byline and abstract of all scientific articles (ie, clinical, basic science, training/education) and case reports published from 2019 to 2021 in the International Journal of Radiation Oncology, Biology, and Physics; Practical Radiation Oncology; and Advances in Radiation Oncology were reviewed. Characteristics of MSs and senior authors are reported.

**Results:**

A total of 105 of 1785 articles (5.8%) included an MS author, among which 72 (68.6%) were clinical, 13 training/education (12.4%), 12 case reports (11.4%), and 8 basic science (7.6%). MS authors were more common for publications in Advances in Radiation Oncology (9.0%) than Practical Radiation Oncology (6.4%) or the International Journal of Radiation Oncology, Biology, and Physics (4.2%; *P* = .002). There were 125 unique MS authors from 72 institutions, among which 40 were first author (32.0%), 28 second author (22.4%), and 57 third (or higher) author (45.6%). There were 88 unique senior authors from 55 institutions, among which 10 (11.3%) were on 2 or more MS publications, and 57 (64.7%) shared the same institution as the MS. The median number of articles per mentor institution was 1 (interquartile range, 1-2), and the mentor institutions in the upper quartile in terms of number of MS publications accounted for 53 (50.5%) of all MS publications.

**Conclusions:**

Few publications in American Society for Radiation Oncology journals include MS authors with mentorship disproportionately from a small number of academic faculty at select institutions. These findings suggest that there is great potential for radiation oncologists to proactively engage more students in their work.

## Introduction

United States (US) medical students (MSs) receive little or no exposure to radiation oncology (RO) during traditional medical school curricula.^1^, ^2^, ^3^, ^4^ A variety of factors may contribute to this, including RO being a small specialty that is infrequently a required component of the curriculum and minimally tested on medical licensing examinations. As a consequence, many students may not have the opportunity to explore the field or consider a career in RO. Therefore, it is important to explore novel and alternative opportunities to engage students in the field, both for career exploration as well as to provide a basic understanding of the role of a radiation oncologist in patient care.^5^ Offering adequate research opportunities to students is one important avenue of doing so, as mentored research often stimulates students’ interest in a particular field and helps them to be competitive residency applicants.^6^, ^7^, ^8^, ^9^ Furthermore, MSs who themselves are interested in scientific research have, historically, been attracted to RO given its emphasis on technology and innovation.^10^, ^11^, ^12^, ^13^

Many radiation oncologists at academic medical centers conduct research; however, the extent to which radiation oncologists directly engage MSs in their research is unknown. Although radiation oncologists publish in many different medical journals, the majority of publications in the American Society for Radiation Oncology (ASTRO) journals derive from the work of radiation oncologists, radiation biologists, or medical physicists. Better characterizing the extent of MS involvement in these publications may offer some indication of the available capacity for academic radiation oncologists to engage students in their research to a greater extent in the future.

## Methods and Materials

This study involved a retrospective review of all byline and abstracts in the International Journal of Radiation Oncology, Biology, and Physics (IJROBP); Practical Radiation Oncology (PRO); and Advances in Radiation Oncology (ARO) from 2019 to 2021. The subsequent analysis only included scientific articles that the journals categorized as clinical, basic science, training/education, and case reports. Article types that were excluded from analysis included review, editorial, clinical guidelines, and correspondence articles. MS authors were defined as individuals affiliated with a medical school but lacking an MD, DO, or PhD degree in the byline. Anytime an individual met our criteria for inclusion as a student based on their degrees but not their affiliation, a web search was conducted using Google, Scopus, Pubmed, and/or LinkedIn to determine their occupation and confirm whether they were an MS (this resulted in exclusion of 12 individuals). The primary faculty mentor was defined as the senior (last) author.

For each journal, the total number of articles in the included categories and the number that included at least 1 MS author was determined. For each article that included an MS author, the article type, MS affiliation and byline position (ie, first, second, third author), senior author affiliation, and total numbers of authors were recorded. For the senior authors, any listed affiliation at a clinical site that was affiliated with a medical school in the same city was considered part of that medical school for the purpose of this analysis. Based on this information, the total number of individual MSs and senior authors as well as the number of articles generated per senior author institution were calculated. PubMed and SCOPUS were used to determine the total number of publications per senior author through the end of 2021. Descriptive statistics are reported. The χ^2^ test was used to assess for differences in the total number of MS articles across the 3 journals.

## Results

A total of 1785 scientific articles were published in all 3 journals over the 3-year period. Among these articles, 105 (5.8%) included at least 1 MS author. Thirty-six (34.2%) were published in 2019, 35 (33.3%) in 2020, and 34 (32.3%) in 2021. The median number of authors for these publications was 6 (interquartile range [IQR], 6-12), with 40 MS first authors (32.0%), 28 MS second authors (22.4%), and 57 MS third (or higher) authors (45.6%). The percentage of scientific articles containing at least 1 MS author was significantly more common (*P* = .002) for publications in ARO (n = 42/468; 9.0%) compared with PRO (n = 21/327; 6.4%) and IJROBP (n = 42/990; 4.2%). Table 1 shows a breakdown of the types of scientific articles containing an MS author in each journal. Clinical articles were most common (n = 72), among which 49 (68.1%) used retrospective research methods.

**Table 1:** Types of scientific article types in each journal with a medical student author

Article type	International Journal of Radiation Oncology, Biology, and Physics (n = 42)	Practical Radiation Oncology (n = 21)	Advances in Radiation Oncology (n = 42)	Total (n = 105)
Clinical	29 (69.1%)	16 (76.2%)	27 (64.3%)	72 (68.6%)
Case report	0 (0%)	2 (9.5%)	10 (23.8%)	12 (11.4%)
Training and education	7 (16.7%)	2 (9.5%)	4 (9.5%)	13 (12.4%)
Basic science	6 (14.3%)	1 (4.8%)	1 (%)	8 (7.6%)

Among the 105 articles that included at least 1 MS author, there were a total of 125 unique MS authors from 72 institutions and 88 unique senior authors from 55 institutions. Additional demographics of students and mentors are shown in Table 2. The median number of total publications of the senior authors was 14 (IQR, 6-29).

**Table 2:** Demographics of medical students and senior authors

Characteristic	Medical students (n = 125)	Senior authors (n = 88)
Sex		
Male	76 (60.8%)	55 (62.5%)
Female	49 (39.2%)	33 (37.5%)
Degree		
MD or DO	In progress	76 (86.4%)
PhD	1 (0.8%)	26 (29.5%)
Masters	16 (12.8%)	15 (17.0%)
RN	0 (0%)	1 (1.1%)
Affiliated with NCI-designated cancer center		
Yes	73 (58.4%)	71 (80.7%)
No	52 (41.6%)	17 (19.3%)
Academic rank		
Professor	-	28 (31.8%)
Associate professor	-	32 (36.4%)
Assistant professor	-	26 (29.5%)
Resident	-	2 (2.3%)

*Abbreviation:* NCI = National Cancer Institute.

Fifty-seven of the senior authors (64.7%) shared the same affiliation as the MS on a given publication. Ten individuals were the senior author for multiple publications that included an MS author, including 6 who published 2 MS articles, 2 who published 3 MS articles, 1 who published 4 MS articles, and 1 who published 5 MS articles. The median number of articles per senior author institution was 1 (IQR, 1-2), and the institutions in the upper quartile in terms of number of MS publications accounted for 53 of all MS publications (51%). The number of publications from institutions with the largest number of MS authors are shown in Table E1.

## Discussion

Offering MSs research in RO provides in-depth exposure to the specialty, which may promote greater interest in pursuing RO as a future career, and insight into the role of RO in patient care. However, this study has demonstrated that only approximately 6% of scientific articles in ASTRO journals from 2019 to 2021 include an MS author. Furthermore, despite the approximately 1800 RO faculty at US MD-granting medical schools,^14^ only 88 served as a senior author in an ASTRO journal for a project that an MS was a coauthor on. Finally, a disproportionate amount of mentorship came from specific individuals and institutions, which likely contributes to the great variability in the number of MSs that pursue RO across US medical schools.^15^ Although not all research is amenable to student involvement, and many radiation oncologists publish in medical journals besides the ones evaluated in this study, our findings suggest that there is great potential for radiation oncologists to proactively engage more students in their work.

To our knowledge this is the first study to specifically evaluate MS authorship of scientific research in RO journals. Prior studies have more broadly evaluated student authorship in IJROBP, as well as other less subspecialized medical journals, reporting student authorship rates ranging from 44% to 58% per year.^16^^,^^17^ However, the methods used in these analyses differed substantially from ours, in that they were not specific to MSs and included undergraduates, masters, and PhD students. Furthermore, they also included published abstracts, which tend to have more MS involvement. Of note, student participation in research was not a disadvantage to scholarly impact (ie, H-index) for the works published in each journal.^16^^,^^17^

Historically RO has been a competitive specialty, and the need for research experience to be competitive in the match may have motivated students to pursue research or clinical opportunities.^8^^,^^18^ In recent years, however, substantially fewer US MSs have applied for RO residency positions,^13^ and it may be necessary for radiation oncologists to take a more proactive approach in attracting high quality and diverse students.^5^ Mentoring students requires effort, and it is critical that a department's culture supports these efforts by providing individual radiation oncologists with adequate time, resources, and encouragement to facilitate their work with students. It is also important that student mentorship be a shared responsibility; for instance, if every academic radiation oncologist offered 1 project per year to an MS, there would be more than enough opportunities for every interested student in the country. Having residents directly mentor students on projects that an attending oversees can also help offset the time requirements for all involved. Examples of proactive approaches that departments might take include advertising available RO projects to the many students seeking research during the summer between their first and second years (most medical dean's offices have a means of doing so), informing students of national opportunities for funded oncology research, or providing departmental funding for student research or meeting presentations. Having a dedicated leader (beyond the clerkship director) to holistically promote MS engagement can also help provide an organizational structure to a department's efforts. It is also notable that although approximately 55% of matriculating medical students are female,^19^ only 39% of the publications in this study were by female medical students and 37% by female senior authors. These numbers approximately mirror the current gender gap in the RO academic workforce.^20^ Given that racial/ethnic or gender concordance of the student-mentor relationship has been shown to lead to greater productivity,^21^^,^^22^ efforts in recruitment of diverse faculty to academic RO departments may also help attract more diverse students to participate in RO research. Finally, because many medical schools lack RO departments, there may be some role for national RO societies to help match interested students with attendings who can offer cross-institutional research projects to motivated students. It was beyond the scope of this study to determine why some specific individuals and institutions contribute to more MS research mentorship, though an assessment of the strategies they use should be the topic of future investigation.

There are several important limitations of this study. First, we did not directly confirm with each MS and senior author that we identified them correctly using our methods, and as such, some may have been categorized incorrectly. Our methods also precluded collection of other relevant demographics of the students or mentors, like race or ethnicity. Additionally, our approach may have missed some publications that were started as MSs but not completed until after an MS graduated and received their MD, DO, or PhD degree. Furthermore, some MS research opportunities may not lead to publications, and, thus, our findings underestimate the total number of research experiences that MSs have with radiation oncologists. Although our data included years before and during the COVID-19 pandemic, we do not believe that the pandemic affected our findings because the number of MS publications was similar in each year evaluated. Finally, we acknowledge that many MSs may participate in research with radiation oncologists published in other journals, though this would not affect our findings related to the ASTRO journals.

## Conclusion

Over the past 3 years, a small fraction of publications in ASTRO journals included MS authors with mentorship disproportionately from a small number of academic faculty at select institutions. Though RO faculty may mentor MSs on projects published in other journals, efforts to more broadly encourage academic RO faculty to offer MSs research opportunities on RO-related projects may help stimulate more interest in RO and reverse the recent trend of fewer residency applicants.

## Disclosures

Malcolm D. Mattes receives grant funding from the Radiation Oncology Institute and Bristol Myers Squibb Foundation, and New Jersey Health Foundation. All other authors have no disclosures to report.

## Appendix Supplementary materials

Supplementary Tables **Supplementary Table 1.** Characteristics of institutions in the upper quartile for MS publications Image, application 1

## Footnotes

Supplementary material associated with this article can be found in the online version at doi:10.1016/j.adro.2023.101323.
