# Supplementary material for: Assessment of Medical Student Research Mentorship in Radiation Oncology
Source: Adv Radiat Oncol. 2023 Jul 23;9(1):101323. doi: 10.1016/j.adro.2023.101323 (PMC10801644; doi:10.1016/j.adro.2023.101323)
Supplement: Supplementary Tables — Supplementary Table 1. Characteristics of institutions in the upper quartile for MS publications [file mmc1.docx]

**Supplementary Table 1.** Characteristics of institutions in the upper quartile for MS publications

| Institution | Number of Publications with Medical Students (n) | Number of Medical Student Authors (n) | Publications from students who shared same institution as senior author (n) | Publications from students from different institution as senior author (n) |
| --- | --- | --- | --- | --- |
| Harvard University | 7 | 8 | 7 | 1 |
| University of Chicago | 6 | 6 | 5 | 1 |
| University of Southern California | 6 | 7 | 2 | 5 |
| Emory University | 5 | 6 | 1 | 5 |
| Stanford University | 4 | 6 | 2 | 4 |
| University of Miami | 4 | 12 | 8 | 4 |
| University of Texas, MD Anderson | 3 | 3 | 2 | 1 |
| Mayo Clinic | 3 | 3 | 2 | 1 |
| University of California, Los Angeles | 3 | 3 | 3 | 0 |
| University of Mississippi | 3 | 1 | 1 | 0 |
| University of South Florida | 3 | 5 | 4 | 1 |
| University of Washington | 3 | 3 | 2 | 1 |
| Washington University in St. Louis | 3 | 3 | 1 | 2 |
| **Total** | **53** | **66** | **40** | **26** |
